# Clinicopathological study of a dimorphic variant of breast carcinoma

**DOI:** 10.1007/s12282-017-0804-x

**Published:** 2017-09-30

**Authors:** Nozomi Ueno, Hajime Kuroda, Masafumi Kurosumi, Yuji Kozuka, Jun Ito, Hiroyuki Kato, Keiichi Kubota, Yasuo Imai

**Affiliations:** 1 Department of Surgery I, Dokkyo Medical University, Mibu, Japan; 2 Department of Diagnostic Pathology, Dokkyo Medical University, 880 Kitakobayashi, Mibu, Tochigi 321-0293 Japan; 3 Department of Pathology, Saitama Cancer Center Hospital, Saitama, Japan; 4 Department of Pathology, Mie University, Tsu, Japan; 5 Department of Surgery II, Dokkyo Medical University, Mibu, Japan

**Keywords:** Dimorphic cell, Breast carcinoma, Low-grade tumor

## Abstract

**Background:**

Dimorphic cells have abundant clear cytoplasm similar to myoepithelial cells, and the nuclei are identical to those in adjacent malignant columnar epithelial cells. A dimorphic variant of a breast carcinoma involves a neoplastic proliferation of epithelial cells including dimorphic cells.

**Methods:**

The subjects were patients with primary breast carcinoma, who underwent surgical resection at the Hospital of Dokkyo Medical University between 2000 and 2016, and were reviewed and diagnosed with a dimorphic variant of breast carcinoma.

**Results:**

Dimorphic ICs typically showed a low-grade tumor and Hormonal receptor (HR) (estrogen and/or progesterone)+/HER2− subtype. Age, mean tumor size, status of nodal metastasis, stage and disease-free survival and overall survival did not differ between dimorphic and non-dimorphic ICs. The dimorphic cells were negative for p63 and cytokeratin 5/6 and 14 in most cases. In contrast, dimorphic cells were positive for HR, androgen receptor, and showed marked membrane-associated staining for E-cadherin and cytoplasmic staining for gross cystic disease fluid protein 15.

**Conclusions:**

The morphological features of dimorphic cells may be confused with cells of other origins if the features of the dimorphic cells are not recognized. However, the typical morphological architecture of this carcinoma and expression of immunohistochemical markers support the diagnosis.

## Introduction

Lefkowitz et al. reported 20 cases of intraductal papillary carcinomas (IPCs) with cuboidal cells with abundant clear or faintly eosinophilic cytoplasm [1–4]. These cells were located mainly near the basement membrane singly, in small clusters, or in broad sheets. The appearance of the polygonal cells was in contrast to the adjacent malignant columnar epithelial (AMCE) cells, and similar to the appearance of the myoepithelium. Lefkowitz et al. suggested that the presence of these tumor cells might create a problem in the differential diagnosis of an IPC as they might be misinterpreted as myoepithelial cells. Despite the difference in cytoplasmic features, the nuclei resemble those in AMCE cells. Because of the variable appearance of these cells, they were designated as dimorphic cells. The biological behavior of this dimorphic variant of ductal carcinoma in situ (dimorphic DCIS) and the precise histogenetic origin of dimorphic cells remain uncertain. The best terminology to describe dimorphic DCIS is a matter of debate [2]. Furthermore, there are dimorphic cells in invasive breast carcinomas similar to DCIS. In this study, we named these carcinomas as a dimorphic variant of invasive carcinoma (dimorphic IC). We herein describe dimorphic variants of breast carcinoma (dimorphic IC and DCIS) and present the clinicopathologic and immunohistochemical characteristics of these tumors.

## Patients and methods

**Fig. 1:**
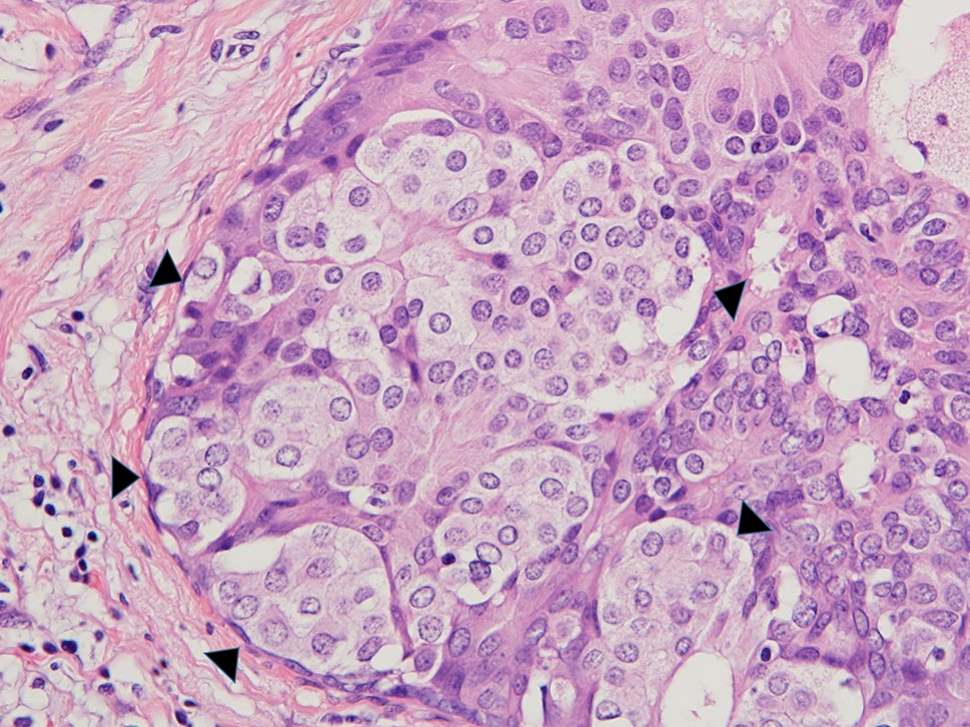
Dimorphic IC on a low-power view (hematoxylin–eosin stain). Typical microscopic appearance of dimorphic cells (hematoxylin–eosin stain). A cluster of tumor cells with faint cytoplasm (dimorphic cell: filled triangle) forming in front of the tumor cells, with adjacent malignant columnar epithelial cells with eosinophilic cytoplasm on a high-power view

**Fig. 2:**
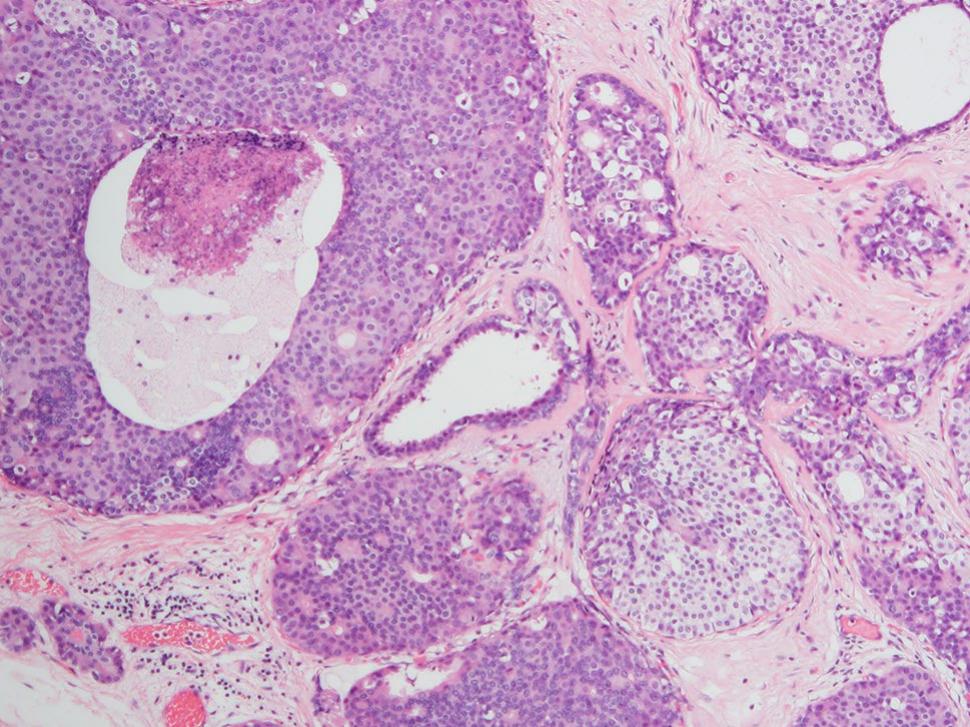
Dimorphic DCIS on a low-power view (hematoxylin–eosin stain). The finding of typical DCIS architecture, such as a cribriform pattern and comedo, supports categorization as DCIS

**Fig. 3:**
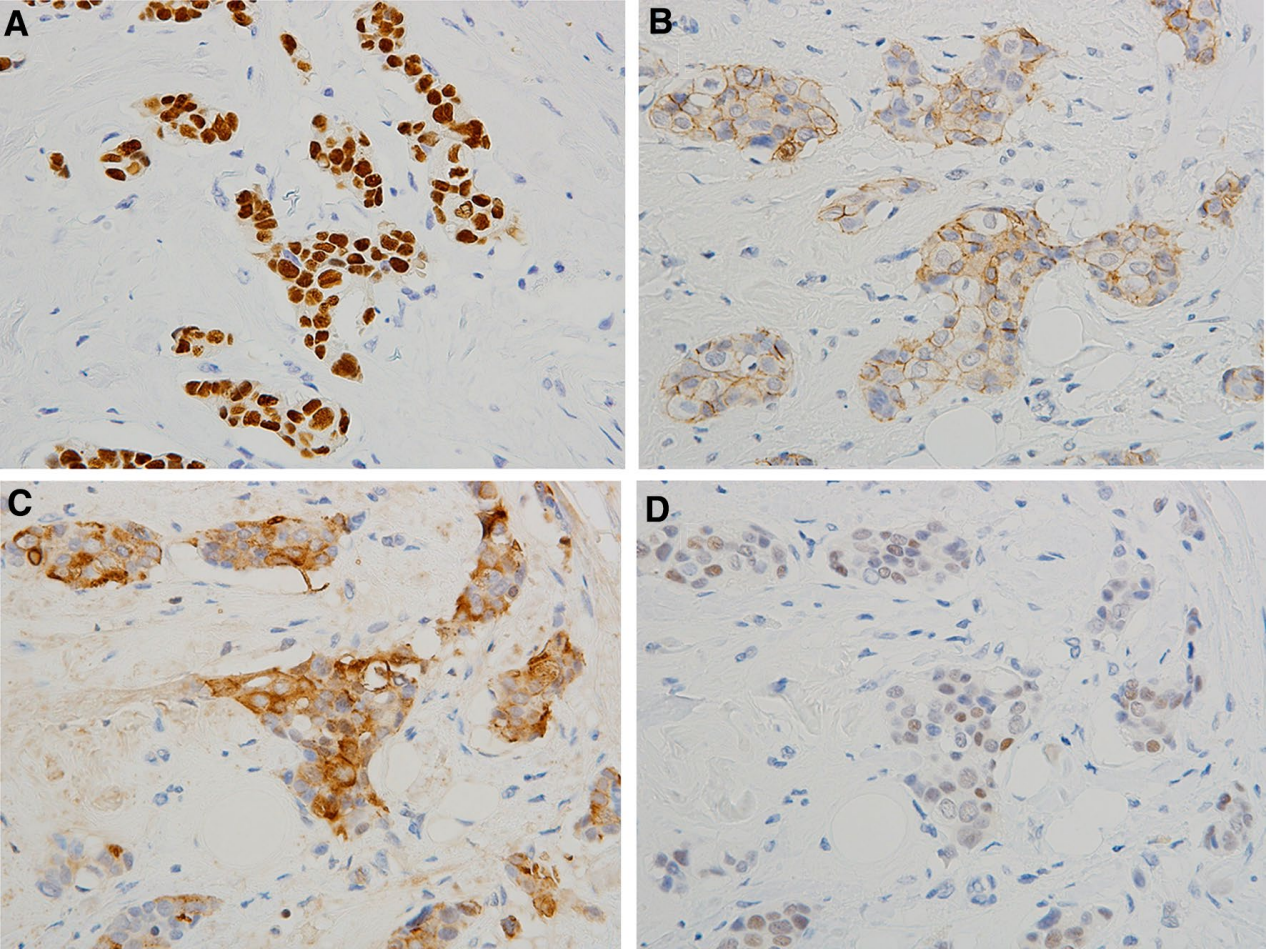
Immunohistochemistry for: **a** estrogen receptor, **b** E-cadherin, **c** gross cystic disease fluid protein 15, and **d** androgen receptor in dimorphic IC

The subjects were cases of primary breast carcinoma that were surgically resected at the Hospital of Dokkyo Medical University (HDMU) between 2000 and 2016, and were reviewed for the histological presentation of dimorphic breast carcinoma. We excluded special types of invasive carcinoma from our cases for comparison. According to the diagnostic criteria of Lefkowitz et al., dimorphic breast carcinoma was defined as the complete presence of dimorphic cells somewhere in carcinoma (Figs. 1, 2, 3) [4]. We also defined dimorphic cells as those that had abundant clear or faintly eosinophilic cytoplasm similar to that seen in myoepithelial cells, the nuclei were identical to those in AMCE cells, and apocrine differentiation such as obvious granular or eosinophilic cytoplasm was not observed anywhere in the tumor.

Patients’ clinical information, including age, mean tumor size, histology, status of lymph node metastasis, disease stage, and clinical outcome were retrieved from their medical records. Clinical outcome was also documented. For each case, all available hematoxylin and eosin-stained sections were reviewed to confirm the diagnosis of mammary disease with no knowledge of prior histological results or clinical outcomes.

### Immunohistochemistry

The antibodies used are summarized in Table 1. The sections were immunostained for estrogen receptor (ER, clone SP1, VENTANA, prediluted, nuclear), progesterone receptor (PgR, clone 1E2, VENTANA, prediluted, nuclear), human epidermal growth factor receptor 2 (HER2, clone 4B5, VENTANA, prediluted, membranous), p63 (p63, clone 4A4, DAKO, 1:50, nuclear), E-cadherin (E-cadherin, clone 36, BD Transduction Lab., 1:2000, Membranous), cytokeratin 5/6 (CK5/6, clone D5/16 B4, DAKO, 1:25, cytoplasmic), cytokeratin 14 (CK14, clone LL002, Novocastra, 1:20, cytoplasmic), androgen receptor (AR, clone AR441, DAKO, 1:50, nuclear), and gross cystic disease fluid protein 15 (GCDFP-15, clone 23A3, Novocastra, 1:40, cytoplasmic). The sections were then placed in an automated stainer (VENTANA, BENCHMARK XT) in accordance with the vendor’s protocol for ER, PgR, and HER2. Other immunohistochemical staining was performed in accordance with the following protocol. Consecutive sections from formalin-fixed, paraffin-embedded tissue blocks were cut at 5 μm intervals, deparaffinized, and dehydrated with xylene and graded alcohol. The slides were treated with methanol containing 0.3% hydrogen peroxide to block any endogenous peroxidase activity. Antigen retrieval was achieved with microwave treatment for all markers. After incubation with the primary antibody, incubation with a secondary, biotinylated antibody was performed for 15 min. After washing, the sections were incubated with streptavidin–peroxidase for 20 min. Finally, the enzyme was visualized after a 5-min incubation with diaminobenzidine. Counterstaining was performed with hematoxylin. The immunohistochemically stained slides of each tumor were compared with positive and negative controls. ER and PgR assays were considered positive if there were at least 1% positive tumor nuclei [5]. The pathologic HER2 status of an invasive carcinoma was defined in accordance with the ASCO/CAP guideline [6]. Hormonal receptor positivity (HR+) was defined as ER+ and/or PgR+, and HR- as both ER- and PgR-. Thus, the four breast carcinoma subtypes were classified as follows: HR+/HER2−, HR+/HER2+, HR−/HER2−, and HR−/HER2+.

**Table 1:** List of antibodies used in this study

No.	Marker	Clone	Source	Dilution	Staining pattern
1	ER	SP1	VENTANA	Prediluted	Nuclear
2	PgR	1E2	VENTANA	Prediluted	Nuclear
3	HER2	4B5	VENTANA	Prediluted	Membranous
4	P63	4A4	DAKO	1:50	Nuclear
5	E-cadherin	36	BD Transduction Lab.	1:2000	Membranous
6	CK 5/6	D5/16 B4	DAKO	1:25	Cytoplasmic
7	CK 14	LL002	Novocastra	1:20	Cytoplasmic
8	AR	AR441	DAKO	1:50	Nuclear
9	GCDFP-15	23A3	Novocastra	1:40	Cytoplasmic

*CK* cytokeratin, *ER* estrogen receptor, *PgR* progesterone receptor, *AR* androgen receptor, *GCDFP-15* gross cystic disease fluid protein-15

### Statistical analysis

*χ* ^2^ analysis or Fisher’s exact test was used to test the association of diagnostic markers with categorical clinicopathological parameters and other biomarkers. Overall and disease-free survival curves were generated according to the Kaplan–Meier method. The differences between the curves were assessed using the log-rank test. A *P* value of < 0.05 was considered statistically significant.

## Results

### Dimorphic IC

Details of the clinicopathological features of the dimorphic ICs are summarized in Table 2. A total of 40 cases of dimorphic ICs and 660 cases of non-dimorphic ICs were reviewed as the control. We excluded special types of invasive breast carcinoma from our study. The patients’ ages ranged from 40 to 77 years (mean 55.4 years) and the mean tumor size ranged from 0.4 to 3 cm (mean 1.4 cm at maximum diameter). Among all cases, grade I occurred in 26 (65.0%) cases, grade II in 13 (32.5%) cases, and grade III in one (2.5%) case. None contained PAS-positive and d-PAS-labile granules in the cytoplasm of tumor cells. Forty patients underwent lymph node dissection. Among these cases, positive nodes occurred in 13 (32.5%) cases, with the number of positive nodes ranging from one to nine (mean 2.6 nodes). Pathologic stage at presentation, according to the American Joint Committee on Cancer staging system, included stage I in 24 (60.0%) cases, stage II in 11 (27.5%) (IIA in eight cases and IIB in three), and stage III in five (12.5%) cases (IIIA in three cases, IIIB in two). Complete five-year follow-up data were available for 29 of the 40 patients diagnosed with dimorphic ICs. Of the 29 patients, local recurrence only occurred in one patient (skin) at 24 months, and all were alive with disease at the latest follow-up.

**Table 2:** Clinicopathologic findings in patients with dimorphic IC and non-dimorphic IC

	Dimorphic IC (40 cases)	Non-dimorphic IC (660 cases)	*P* value*
Mean age	55.4 (40 to 77)	58.1 (27 to 93)	0.2381
Mean tumor size (cm)	1.4 (0.4 to 3)	1.6 (0.1 to 7.2)	0.0586
Histological grading			
I	26 (65.0%)	254 (38.5%)	<0.001*
II	13 (32.5%)	265 (40.1%)	
III	1 (2.5%)	141 (21.4%)	
Status of nodal metastasis	13 of 40 (32.5%)	237 of 633 (35.9%)	0.5305
Stage			
I	24 (60.0%)	324 (49.1%)	0.4545
II	11 (27.5%)	221 (33.5%)	
III	5 (12.5%)	95 (14.4%)	
IV	0 (0%)	20 (3.0%)	
5-year follow-up data available cases	29 of 40	465 of 660	
Recurrence	1 (3.5%)	50 (10.8%)	0.2461**
Local recurrence	1 (3.5%)	4 (0.9%)	
Distant metastasis	0 (0%)	46 (9.9%)	
Died of disease	0 (0%)	23 (4.3%)	0.2804**
HR+/HER2−	37 (92.5%)	474 (71.8%)	0.0042*
HR+/HER2+	1 (2.5%)	40 (6.1%)	0.5589
HR−/HER2−	0 (0%)	98 (14.9%)	0.0086*
HR−/HER2+	2 (5.0%)	48 (7.2%)	0.8213

*IC* invasive carcinoma, *HR* hormone receptor

* P < 0.05

** Log-rank test

The dimorphic IC expressed ER in 37 (92.5%) cases, PgR in 36 cases (90.0%), and HER2 in three (7.5%) cases. Thirty-seven of 40 dimorphic ICs were classified as HR+/HER2− (92.5%). For the remaining cases, one (2.5%), none, and two (5.0%) cases were classified as HR+/HER2+, HR−/HER2−, and HR−/HER2+, respectively. Dimorphic ICs typically showed, a low-grade tumor and HR+/HER− subtype. Age, mean tumor size, status of nodal metastasis, stage and disease-free survival and overall survival did not differ between dimorphic and non-dimorphic ICs.

The immunohistochemical analyses are summarized in Table 4. The dimorphic cells comprising dimorphic IC were negative for p63, CK5/6, and CK14 in most cases. However, dimorphic cells were diffusely positive for nuclear ER and AR, and showed marked membrane-associated staining for E-cadherin and cytoplasmic staining for GCDFP-15 (Fig. 3a–d). There were no expression pattern differences between the dimorphic cells and AMCE cells.

### Dimorphic DCIS

Details of the clinicopathological features of the dimorphic DCISs are summarized in Table 3. A total of ten cases of dimorphic DCISs and 124 cases of non-dimorphic DCISs were reviewed as the control. The patients’ ages ranged from 41 to 77 years (mean 53.0) and the mean tumor size ranged from 0.5 to 6 cm (mean 1.9 at maximum diameter). Histologically, nine of ten dimorphic DCISs were classified as standard DCIS (90.0%) and one as a solid papillary carcinoma (10.0%). Five patients presented with low grade, three with intermediate grade and two with high grade DCIS. There was no lymph node metastasis. Complete 5-year follow-up data were available for nine of the ten patients. Of these patients, no patient had local or systemic recurrence, or had died of the disease following the initial diagnosis of the primary lesion.

**Table 3:** Clinicopathologic findings in patients with dimorphic DCIS and non-dimorphic DCIS

	Dimorphic DCIS (10 cases)	Non-dimorphic DCIS (124 cases)	*P* value*
Mean age	53 (41–77)	58.2 (27–95)	0.2819
Mean tumor size (cm)			
	1.9 (0.5–6)	1.4 (0.1–10.5)	0.3305
	DCIS 9 (90.0%)	DCIS 99 (79.8%)	0.9068
	Solid papillary 1 (10.0%)	Solid papillary 14 (11.3%)	
Histology		Encapsulated 8 (6.5%)	
		DCIS in sclerosing adenosis 2 (1.6%)	
		DCIS in fibroadenoma 1(0.8%)	
Grading			
Low	5 (50.0%)	56 (45.2%)	0.8286
Intermediate	3 (30.0%)	32 (25.8%)	
High	2 (20.0%)	36 (29.0%)	
Status of nodal metastasis	0 of 10 (0%)	1 of 108 (0.9%)	0.7599
Follow-up data available cases	9 of 10	114 of 124	
Recurrence	0 (0%)	2 (1.8%)	0.6905**
Local recurrence	0 (0%)	1 (0.9%)	
Distant metastasis	0 (0%)	1 (0.9%)	
Died of disease	0 (0%)	0 (0%)	NA
HR+	10 (100.0%)	99 (79.8%)	0.1154

*DCIS* ductal carcinoma in situ, *HR* hormone receptor, *NA* not assessed

* P < 0.05

** Log-rank test

Trends between the age, tumor size, histology, tumor grade, status of nodal metastasis disease-free survival and overall survival were compared with non-dimorphic DCISs. However, there were no significant differences (Table 3).

On a low-power view, the dimorphic DCISs exhibited a typical DCIS pattern. A neoplastic proliferation of epithelial cells composed of two cell patterns, eosinophilic and clear stained cytoplasm (adjacent malignant columnar epithelial cells and dimorphic cells), was observed. Dimorphic cells resembling myoepithelial cells were noted. However, dimorphic cells could be discriminated by their localization difference with myoepithelial cells and similar nuclear morphology with columnar cells.

The immunohistochemical analyses are summarized in Table 4. The two cell types comprising dimorphic DCIS were negative for p63, CK 5/6 and 14 in all cases. However, both cell types were diffusely positive for nuclear ER and AR, as well as marked membrane-associated staining for E-cadherin in all cases. Cytoplasmic staining for GCDFP-15 was observed in eight of the ten cases studied in both cell types.

**Table 4:** The staining pattern of dimorphic breast carcinoma

	Markers	Dimorphic cells	Adjacent malignant columnar epithelial cells	*P* value*
Dimorphic IC				
P63		1 (2.5%)	0 (0%)	0.3143
E-cadherin		39 (97.5%)	39 (97.5)	NA
CK 5/6		0 (0%)	0 (0%)	NA
CK 14		0 (0%)	0 (0%)	NA
ER		37 (92.5%)	37 (92.5%)	NA
AR		35 (87.5%)	35 (87.5%)	NA
GCDFP-15		34 (85.0%)	34 (85.0%)	NA
Dimorphic DCIS				
P63		0 (0%)	0 (0%)	NA
E-cadherin		10 (100.0%)	10 (100.0%)	NA
CK 5/6		0 (0%)	0 (0%)	NA
CK 14		0 (0%)	0 (0%)	NA
ER		10 (100.0%)	10 (100.0%)	NA
AR		10 (100.0%)	10 (100.0%)	NA
GCDFP-15		8 (80.0%)	8 (80.0%)	NA

*CK* cytokeratin, *ER* estrogen receptor, *AR* androgen receptor, *GCDFP-15* gross cystic disease fluid protein-15, *NA* not assessed

* P < 0.05

## Discussion

Lefkowitz et al. reported IPCs with cuboidal cells with abundant clear or faintly eosinophilic cytoplasm that bore a resemblance to myoepithelial cells [4]. Despite the difference in cytoplasmic features between the clear cells and AMCE cells, the nuclei were identical to those in AMCE cells. Because of the variable appearance of these clear cells, they were designated as dimorphic cells. Further, dimorphic cells were located mainly near the basement membrane in dimorphic DCIS. However, previously, there has been no description of the distribution of dimorphic cells in dimorphic ICs. In dimorphic IC, dimorphic cells form a solid mass in the intraductal components in some cases, but are also distributed in diffuse invasive areas. We could not confirm the specific location of dimorphic cells in our small number of dimorphic IC cases. The morphology raises the issue of whether dimorphic breast carcinoma could be diagnosed as a variant of breast carcinoma. However, most cases were previously diagnosed as IC and DCIS, with the observation of typical architecture, and this supports categorization as a variant of breast carcinoma on low-power views. Furthermore, there were no differences in nuclear morphology between the dimorphic cells and AMCE cells in high-power views. Thus, dimorphic breast carcinoma may be an accurate diagnosis on the basis of the architectural pattern and careful observation of the cytological features.

Different morphology also raised the question of whether dimorphic IC has different biological behaviors. Only one patient had local recurrence and no patients died from disease-related complications with dimorphic IC. One explanation for this observation is that dimorphic ICs typically form a low-grade tumor, explaining the good prognosis. Furthermore, immunohistochemistry-based analysis of ER, PgR, and HER2 expression may serve as a surrogate assay or molecular analysis, and the HR+/HER2− subtype is considered to be a luminal type [7–10]. Most dimorphic ICs were classified as the HR+/HER2− (luminal) subtype, which was significantly higher compared with non-dimorphic ICs [1–3]. Moreover, the ratio of HR−/HER2−, which is known as a triple negative carcinoma, was low in dimorphic IC. These findings led us to hypothesize that the tumor had a potentially low degree of aggressiveness. In contrast to these findings, we could not find any significant difference in dimorphic DCISs. However, we could not draw any conclusion about the histological characteristics or prognostic impact based on the small number of dimorphic DCIS.

Lefkowitz et al. reported that smooth muscle actin, S-100 and GCDFP-15 were negative in dimorphic cells in ten of 20 cases studied [4]. However, the precise histogenetic origin of dimorphic cells remains uncertain in breast carcinoma. Recent immunohistochemical markers that can aid in determining accurate origins of dimorphic cells have been identified. The immunohistochemical markers p63, CK5/6, and CK14 proved to be the most sensitive markers for the detection of myoepithelial cells of the breast, and may be helpful for the evaluation of dimorphic cells that resemble myoepithelium [11–14]. Furthermore, recent reports suggested that secretory carcinomas express CK5/6 and CK14 [15, 16]. In our study, despite the differences in cytoplasmic features between eosinophilic and clear or faint stained cytoplasm cells, there was no reactivity for p63, CK5/6, or CK14 in dimorphic cells or AMCE cells in most cases. Furthermore, for the differential diagnosis of lobular carcinoma infiltration, one common immunohistochemical feature was the lack of reactivity for E-cadherin [17, 18]. E-cadherin is a cell–cell adhesion protein fulfilling a prominent role in epithelial differentiation and serving as an aid in the sub-classification of breast carcinoma. We demonstrated in all cases that both cell types were E-cadherin-positive, suggesting non-lobular carcinoma proliferation. In most cases of apocrine carcinoma, the cytoplasm exhibits eosinophilia that may be homogenous or granular. However, some apocrine cells have cytoplasmic vacuolization or clear cytoplasm resembling dimorphic cells whereas other areas display a variable appearance with a mixture of obvious apocrine appearance. In our case, characteristic apocrine differentiation such as obvious granular or eosinophilic cytoplasm was not observed anywhere in the tumor. Moreover, immunohistochemical staining supports the confirmation of dimorphic cells. Apocrine carcinoma is negative for hormone receptors in general. In contrast, most dimorphic breast carcinomas (non-dimorphic IC and DCIS) are hormonal receptor (ER, PgR) diffusely positive. The results for dimorphic cells also reinforce the difference from cancer cells with apocrine metaplasia. Because AR is frequently expressed in apocrine carcinoma and in benign apocrine lesions, we used this marker for differentiating lesions [19–22]. Our cases demonstrated diffuse AR positivity in both cell types. However, there are several reports that breast carcinoma expresses AR through a mechanism independent of apocrine metaplasia. Moinfar et al. reported 87 of 145 cases (60%) of invasive carcinoma, and 45 of the 55 cases (82%) of DCIS were AR-positive by immunohistochemical methods [22]. They also reported a higher incidence of AR expression, especially in Grade I invasive carcinoma and low grade DCIS, as opposed to Grade III invasive carcinoma and high grade DCIS. In our study, most of the dimorphic ICs were Grade I invasive carcinoma and low grade DCIS; this may be associated with a high incidence of AR expression. GCDFP-15 is also regarded as a specific marker of apocrine cells and is strongly expressed in apocrine breast carcinoma [23, 24]. Its expression, however, is not limited to apocrine morphology, as it is also expressed in general breast carcinoma. This again emphasizes the importance of first recognizing the morphological features of the dimorphic cell before interpreting either AR or GCDFP-15 positivity as indicative of apocrine metaplasia or carcinoma. From our results, there were no expression pattern differences between dimorphic cells and AMCE cells, which also led us to speculate that dimorphic cells from the same origin were a variant of invasive breast carcinoma.

In conclusion, this report describes the clinicopathological features, and immunohistochemical marker expression patterns, in dimorphic breast carcinoma. The morphological features of dimorphic cells may be confused with cells of other origins if the features of dimorphic cells are not recognized. The typical morphological architecture of the carcinoma and expression of immunohistochemical markers supported the diagnosis. In our study, the dimorphic IC appeared to have a potentially low degree of aggressiveness. However, the underlying etiology of the biological behavior of dimorphic breast carcinoma is still uncertain. Further studies are necessary to elucidate the mechanism of this unique pattern of breast carcinoma.
